# Quantification of Upper Limb Motor Recovery and EEG Power Changes after Robot-Assisted Bilateral Arm Training in Chronic Stroke Patients: A Prospective Pilot Study

**DOI:** 10.1155/2018/8105480

**Published:** 2018-03-26

**Authors:** Marialuisa Gandolfi, Emanuela Formaggio, Christian Geroin, Silvia Francesca Storti, Ilaria Boscolo Galazzo, Marta Bortolami, Leopold Saltuari, Alessandro Picelli, Andreas Waldner, Paolo Manganotti, Nicola Smania

**Affiliations:** ^1^Neuromotor and Cognitive Rehabilitation Research Centre (CRRNC), Department of Neuroscience, Biomedicine and Movement Sciences, University of Verona, Verona, Italy; ^2^U.O.C Neurorehabilitation Unit, AOUI of Verona, Verona, Italy; ^3^San Camillo Hospital IRCCS, Venice, Italy; ^4^Department of Computer Science, University of Verona, Verona, Italy; ^5^Department of Neurology, Hochzirl Hospital, 6170 Zirl, Austria; ^6^Research Unit for Neurorehabilitation South Tyrol, 39100 Bolzano, Italy; ^7^Department of Neurological Rehabilitation, Private Hospital Villa Melitta, Bolzano, Italy; ^8^Clinical Unit of Neurology, Department of Medical Sciences, University Hospital and Health Services of Trieste, University of Trieste, Trieste, Italy

## Abstract

**Background:**

Bilateral arm training (BAT) has shown promise in expediting progress toward upper limb recovery in chronic stroke patients, but its neural correlates are poorly understood.

**Objective:**

To evaluate changes in upper limb function and EEG power after a robot-assisted BAT in chronic stroke patients.

**Methods:**

In a within-subject design, seven right-handed chronic stroke patients with upper limb paresis received 21 sessions (3 days/week) of the robot-assisted BAT. The outcomes were changes in score on the upper limb section of the Fugl-Meyer assessment (FM), Motricity Index (MI), and Modified Ashworth Scale (MAS) evaluated at the baseline (T_0_), posttraining (T_1_), and 1-month follow-up (T_2_). Event-related desynchronization/synchronization were calculated in the upper alpha and the beta frequency ranges.

**Results:**

Significant improvement in all outcomes was measured over the course of the study. Changes in FM were significant at T_2_, and in MAS at T_1_ and T_2_. After training, desynchronization on the ipsilesional sensorimotor areas increased during passive and active movement, as compared with T_0_.

**Conclusions:**

A repetitive robotic-assisted BAT program may improve upper limb motor function and reduce spasticity in the chronically impaired paretic arm. Effects on spasticity were associated with EEG changes over the ipsilesional sensorimotor network.

## 1. Introduction

Poststroke upper limb impairment strongly influences disability and patients' quality of life [1, 2]. Considering that up to two-thirds of stroke survivors suffer from upper limb dysfunctions, one of the main goals of rehabilitation is to improve recovery of upper limb functioning. Many rehabilitation approaches have been put forward [3–5]. However, there is strong evidence that the conceptual evolution of stroke rehabilitation promotes high-intensity, task-specific, and repetitive training [3, 5, 6]. To this end, the application of robot-assisted therapy has steadily gained acceptance since the 1990s [7, 8]. Robotic devices, in fact, allow repetitive, interactive, high-intensity, and task-specific upper limb training across all stages of recovery and neurological severity as well [6].

A meta-analysis has shown significant, homogeneous positive summary effect sizes (SESs) for upper limb motor function improvements and muscle strength with the use of elbow-wrist robots in a bilateral mode [5]. Although subgroup analysis revealed no significant differences between phases post stroke [5], bilateral arm training (BAT) has shown great promise in expediting progress toward poststroke recovery of upper limb functioning even in the chronic phase [6, 9–11].

BAT is a form of training in which both upper limbs perform the same movements simultaneously and independently of each other [12]. It can be undertaken in different modes (in-phase, antiphase) and training modalities (i.e., active, passive, and active-passive) [13]. The beneficial effects of BAT are thought to arise from a coupling effect in which both limbs adopt similar spatio-temporal movement parameters leading to a sort of coordination [14]. Active-passive BAT of the wrist has been investigated in behavioral and neurophysiological studies [11, 15]. It consists of rhythmic, continuous bimanual mirror symmetrical movements during which the patient actively flexes and extends the “unaffected” wrist, while the device assists the movement of the “affected” wrist in a mirrored, symmetrical pattern via mechanical coupling [15–19]; that is, movement of the affected upper limb is facilitated by the unaffected one [12]. Previous studies have reported that this pattern of coordinated movement leads to improvements in upper limb function [11, 16, 19, 20] associated with an increase in ipsilesional corticomotor excitability [11]. In addition, passive BAT of the forearm and the wrist has been shown to lead to a sustained reduction of muscle tone in hemiparetic patients with upper limb spasticity [20].

Current evidence indicates that the neural correlates of BAT are poorly understood [13]. The limitations of previous studies are threefold. First, patient characteristics such as type and site of stroke lesion were not consistently reported [21], precluding full understanding of motor and neural responses to BAT. Second, different BAT modalities (i.e., in-phase, antiphase, active, and active-passive) combined or not with other interventions (i.e., functional tasks or free movements with rhythmic auditory cues) have been reported. As different training modalities are thought to exploit different clinical effects and neural mechanisms [22], the relationship between each of these specific modes (delivered as a single intervention) and brain activity patterns needs to be more precisely explored [13]. Finally, a wide range and variation of neurophysiological and neuroimaging measures have been used among studies.

Essentially, transcranial magnetic stimulation (TMS) and functional magnetic resonance imaging (fMRI) studies have been used to investigate the neural correlates of BAT. Strength and weakness might be acknowledged for both techniques when applied in a neurorehabilitation setting [23]. TMS is an important tool that fits in the middle of the functional biology continuum for assessment in stroke recovery. However, it has the disadvantage of not being as relevant as other biologic measures in gathering information on brain activity during different states (or tasks) [23], unless electroencephalography (EEG) is recorded simultaneously [24].

Functional imaging and related techniques ((fMRI), positron emission tomography (PET), EEG, magnetoencephalography (MEG), and near-infrared spectroscopy (NIRS)) are important tools to determine the effects of brain injury and how rehabilitation can change brain systems [23]. fMRI is the most widely used technique for studying brain function. Several fMRI studies have described movement-related changes in motor cortical activation during partial recovery of the affected limb in stroke patients [25], and many studies have described the effects of various rehabilitative treatments on motor activation.

fMRI shows difficulties when exploring brain functions during robot-assisted sensorimotor tasks because only a few devices are MRI compatible [26–28] and their use in the clinical setting is limited by regulation (i.e., CE marking).

The EEG technique, conversely, has considerable advantages over other methods in the rehabilitation setting [17, 18, 29] being portable and readily operable with different robotic devices. Finally, the higher temporal resolution of EEG than fMRI signals allows monitoring brain activity during movement execution [30–32]. EEG alpha and beta band powers decrease during motor execution over the premotor and primary sensorimotor cortex; at the end of the movement, a rebound of beta activity is observed over the ipsilesional side. These power changes are termed, respectively, event-related desynchronization (ERD)—that is, power band decrease—and event-related synchronization (ERS)—that is, power band increase [33].

To the best of our knowledge, no study has addressed changes in EEG power alongside changes in upper limb motor function after passive robot-assisted BAT (R-BAT). Therefore, the aim of this pilot study was to evaluate changes in both EEG power by investigating the topographical distribution of event ERD/ERS, and upper limb recovery of function after passive R-BAT in chronic stroke patients. Conducting a small-scale pilot study before the main study can enhance the likelihood of success of the main study. Moreover, information gathered in this pilot study would be used to refine or modify the research methodology and to develop large-scale studies [34]. The work hypothesis was that R-BAT would improve recovery of upper limb function and that these effects would be associated with an increase in activation of the ipsilesional hemisphere.

## 2. Methods

This study was a within-subject design. Seven right-handed male outpatients aged ≥ 18 years with first-ever stroke were recruited. Inclusion criteria were unilateral stroke (hemorrhagic or ischemic) as documented by radiologic evidence; at least 6 months from stroke; Medical Research Council (MRC) scale score ≤ 4 [35] evaluated at the wrist and finger extensors; and motor function stability assessed by means of a 2-week baseline evaluation.

Exclusion criteria were multi-infarctual cerebrovascular pathology; Mini-Mental State Examination (MMSE) score ≤ 24/30 [36]; Modified Ashworth Scale (MAS) score > 4 [37] at the wrist and/or fingers; botulinum toxin injections in the 12 weeks prior to and/or during the study period; presence of metallic implants in the brain; previous brain surgery; use of medications altering cortical excitability or with a presumed effect on brain plasticity; and any other diagnosis having a major effect on upper limb function. Patients were not receiving any type of physical therapy for the affected upper limb during the study period.  Table 1 presents patients' demographic and clinical data.

Control data from eight healthy volunteers (5 women; mean age 26.38 years ± SD 2.62 years), performing the same experimental motor paradigm, were gathered from our previous study [17]. The local ethics committee of the Verona University Department and Hospital (CE number 2366) approved the study. All participants provided written informed consent in accordance with the Declaration of Helsinki.

### 2.1. Assessments

Demographic and clinical data were collected at enrollment. Neurological severity and disability were assessed by the European Stroke Scale [38] and the Barthel Index [39], respectively. The precise site of stroke was identified by lesion mapping analysis using MRIcron software (http://www.mricro.com/mricron) (Table 1). MRI images were gathered from each patient, except for one (number 6) for digital imaging unavailability. Lesions were visually identified as having altered FLAIR signal intensity compared to corresponding contralateral tissue [40]. An expert clinical neurologist confirmed the lesion, and a trained image analyst traced all lesions visually identified using digital T1-weighted anatomical MRI scan. The ch2bet anatomical brain template provided with the MRIcron software was used to draw three-dimensional regions of interest (ROIs) [41]. For each patient, the ROI images were converted into volume of interest (VOI) images using MRIcron software (http://www.mricro.com/mricron). The regions of the brain that have sustained damage were computed and reported in Table 1.

Patients completed baseline assessment with primary and secondary outcome measures. Baseline values were obtained by averaging the baseline scores (T_0_). The Fugl-Meyer motor assessment (FM) was the primary outcome measure [42–44]. It is a sensitive, reliable, and valid test to evaluate functional improvements in stroke studies on robotic upper limb rehabilitation (score range, 0–66; with higher scores indicating better performance) [42, 44]. Patients with a FM score < 18 were considered affected by severe-moderate upper limb paresis [19]. Although the minimal clinically important difference on the FM scale is not yet known in chronic stroke patients, a greater than 10-point (10%) change in FM motor scores was considered a clinically meaningful improvement [45].

The secondary outcomes were the Action Research Arm Test (ARAT) [46, 47] to evaluate upper limb function (score range, 0–57; with higher scores indicating better performance), the Motricity Index (MI) upper limb items (Bohannon et al. 1999) to evaluate upper limb strength (score range, 0–99; with higher scores indicating greater strength), the MAS [37] to evaluate upper limb spasticity (score range, 0–4; with higher scores indicating worse spasticity; total score, 0–16; with higher scores indicating worse spasticity), and the Barthel Index (BI) to evaluate disability.

Clinical assessments were repeated after training (T_1_) and at 1-month follow-up (T_2_). The same therapist, who was unaware of the nature of the study, assessed all patients. All outcome measures after training are expressed in relation to the baseline values.

### 2.2. Neurophysiological Measures and Motor Paradigm

EEG data were acquired using a video-EEG system (Ates Medica Device, Verona, Italy) and a cap (Electrical Geodesic Inc., Eugene, OR, USA) with 32 Ag/AgCl electrodes positioned according to a 10/20 international system. The reference was placed at Cz. The electromyographic (EMG) signal was recorded from the right and left flexor carpi radialis muscles with two surface Ag/AgCl electrodes in a belly tendon montage. This served to trigger the movement onset and to monitor movements required by the tasks (i.e., involuntary mirror movements and any other unspecific muscle activations). The EEG data were acquired at a rate of 250 Hz using the software package Geodesic EEG System on Neurotravel technology (Ates Medica Device and Electrical Geodesic Inc.).

The video-EEG was performed during a robot-assisted motor paradigm using the Bi-Manu-Track (BMT) (Reha-stim Co, Berlin, Germany) [19, 20]. The protocol consisted of six tasks involving unilateral, bilateral, passive, and active movements of wrist flexion/extension [17, 18, 29]. In each protocol, six 20 s runs of rest alternating with six 20 s runs of execution were performed. Task execution was acoustically paced with a metronome at a frequency of 1 Hz. The metronome ticking continued during both activation and rest blocks to keep input constant. The subject was signaled to start and stop the task according to the experimenter's vocal instruction “start” and “stop,” respectively. To perform the task correctly, each subject was trained for several minutes before the experiment.

EEG assessments were carried out at T_0_, T_1_, and T_2_ during one-day experimental sessions in a quiet environment in the afternoon. The data were processed in MATLAB 7 (MathWorks, Natick, MA, USA) using scripts based on EEGLAB toolbox (Delorme and Makeig 2004), as well as a custom-made code created for this study. The EEG recordings were band-pass filtered from 1 to 30 Hz; visible artifacts were removed using an independent component analysis procedure [48], and data were processed using a common average reference. EMG signal was band-pass filtered from 10 to 450 Hz and rectified. The envelope was computed by low-pass filtering the signal (5 Hz). The threshold level for activity was identified for EMG by measuring the standard deviation of the signal during rest condition. The threshold level was set at two times this standard deviation. The time of EMG offset was identified as the intersection of the envelope signal with the threshold level. The EEG data of each rest and active run, selected by EMG signal, were divided into epochs of 2 s. Power spectral density (*μ*V^2^/Hz) was estimated using a fast Fourier transform (FFT) applied to 2 s period and then averaged separately for each condition (rest and active). The recordings were Hamming-windowed to control for spectral leakage. A ERS/ERD procedure was used to quantify the changes in EEG power in the upper alpha (10–12 Hz) and the beta (13–30 Hz) frequency ranges. ERD/ERS transformation was defined as the percentage decrease/increase of power density during the task with respect to the baseline value (rest condition). Event-related power decreases, which represent a decrease in synchrony of the underlying neuronal populations and indicate cortical activation state, are expressed as negative values, whereas event-related power increases indicating a cortical idling state are expressed as positive values [33, 49]. The alpha and beta ERD/ERS maps were derived for all patients and used for individual analysis.

A laterality index (LI) [50], describing the contrast in amount of activation (i.e., ERD in alpha and beta bands) between the right and left hemisphere, was calculated during all movement tasks according to

(1) $$\begin{array}{c} LI=\left\{\begin{array}{ll} \frac{\left(\left|{ERD}_{C}\right|-\left|{ERD}_{I}\right|\right)}{\left(\left|{ERD}_{C}\right|+\left|{ERD}_{I}\right|\right)} & \text{if }{ERD}_{C}<0\text{ and }{ERD}_{I}<0,\\ 0 & \text{if }{ERD}_{C}>0\text{ and }{ERD}_{I}>0,\\ +1 & \text{if }{ERD}_{C}<0\text{ and }{ERD}_{I}>0,\\ -1 & \text{if }{ERD}_{C}>0\text{ and }{ERD}_{I}<0, \end{array}\right. \end{array}$$

where ERDc is the C3 ERD value over the contralateral (left) sensorimotor (SM) area and I is the C4 ERD over the ipsilateral (right) SM area. LI can thus range from +1 (exclusively contralateral) to −1 (exclusively ipsilateral).

### 2.3. Intervention

The training consisted of twenty-one 50-minute individual sessions, 3 days/week (Monday, Wednesday, and Friday), for 7 consecutive weeks. The BMT is a robotic arm trainer that allows bimanual practice of 1 degree of freedom pronation and supination movements of the forearm and the wrist in dorsiflexion and volarflexion [19, 20]. It has been designed to train distal movements (elbow and wrist), which are an integral part of activities of daily living. The patients sat at a height-adjustable table with their elbows flexed at 90° and their forearms inserted in an arm trough in the midposition. Each hand grasped a handle. A strap kept the affected hand in place. The training protocol consisted of 5 minutes of upper limb mobilization and stretching exercises to enhance flexibility and facilitate positioning on the BMT, followed by 45 minutes of robot-assisted BAT. Two computer-controlled modes were trained. The passive-passive modes consisted of smooth passive movements driven by the robotic system at a preselected speed and range of motion (ROM). The passive mode is an accepted mobilization technique in the neurodevelopmental framework to improve joint, muscle, and tendon flexibility, as well as to reduce muscle tone [51]. In addition, functional imaging and neurophysiological studies have shown that passive hand movements in healthy controls and in stroke patients resulted in a similar activation of the corresponding sensorimotor cortical areas [17, 18, 52–54]. The active-passive mode consisted of passive movements of the affected upper limb driven by the unaffected side. It is an accepted rehabilitation technique with a facilitatory effect on the affected upper limb, as previously discussed.

During each session, the patients performed up to 800 repetitions as follows: 400 repetitions in passive-passive mode (200 prono/supination and 200 wrist flexion/extension) and 400 in active-passive mode (200 prono/supination and 200 wrist flexion/extension). One repetition included both movement directions (forearm prono/supination and wrist flexion/extension). Treatments were performed in the same room by the same therapists not involved in the assessment procedures. The therapists set up the device and remained within shouting distance in case of any problems. Training parameters (i.e., ROM, movement speed, and number of repetitions) were tailored to each patient's ability and progressively increased as the patient improved. The therapists recorded on the patient's chart the exercises performed during each treatment session, improvements observed, and any adverse events occurring during the study period. During the study, patients did not receive other rehabilitation treatments than scheduled in the current protocol. They were allowed performing activities of daily function and physical activity according to their upper limb function. No advices on home-based exercises or any other upper limb training were given.

### 2.4. Statistical Analysis

Descriptive statistics included mean, standard deviation, and median. Brodmann areas and white matter tracts involved from the brain damage were reported in decreasing order according to the number of involved voxels in Table 1. Coloured maps representing the brain lesion were generated onto the automated anatomical labeling (AAL), and the Johns Hopkins University white matter templates provided *i*th MRIcron software [41] and displayed in figures. Shapiro-Wilk and Levene tests were used to check the normality of distribution and the homogeneity of variance. Because clinical data gathered from clinical scales are not normally distributed, nonparametric tests were applied. The Friedman test was used to analyze changes in performance over time. Post hoc comparisons were carried out on differences in T_0_-T_1_ and T_0_–T_2_ scores using Wilcoxon signed-ranks tests.

Because the EEG data were normally distributed, parametric tests were used. Comparison of the control group versus each single patient was carried out using a *z*-test (*p* < 0.1). In detail, the statistical map defines the electrodes in which ERD/ERS values from a patient differ statistically from those of a reference population (control group). To compare patients and the reference population, which was acquired with only 21 EEG channels, 19 electrodes out of 30 were included in the statistical analyses (Fp1, Fp2, F7, F3, Fz, F4, F8, T3, C3, Cz, C4, T4, T5, P3, Pz, P4, T6, O1, and O2). A three-way analysis of variance (ANOVA) for repeated measures was applied to the relative power with the three factors: “time” (T_0_, T_1_, and T_2_), “task” (unimanual and bimanual active and passive movements), and “channel” (19 electrodes). The sphericity assumption was assessed using Mauchly's test, and Greenhouse Geisser corrections were undertaken when sphericity was violated. Statistical results were deemed significant if *p* < 0.05. A post hoc two-tailed *t*-test adjusted for multiple comparisons with the Bonferroni method was used whenever appropriate. Finally, the Pearson correlation coefficient was used to assess the relationships between LI and clinical scales (FM and MAS), and between ERD/ERS values and clinical scales (FM and MAS) at each evaluation time point (T_0_, T_1_, and T_2_). *p* < 0.05 was considered statistically significant. Software statistics IBM SPSS (20.0) for Macintosh software (IBM, Armonk, NY, USA) was used.

## 3. Results

**Figure 1:**
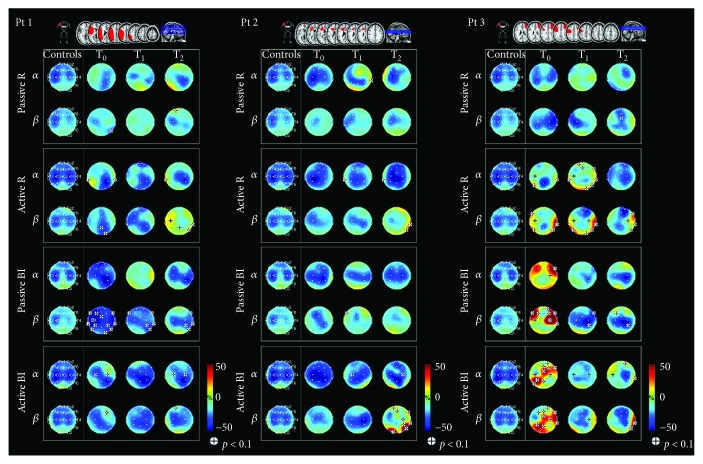
Lesions displayed on a magnetic resonance imaging brain template and topographic maps showing ERD/ERS values. ERD/ERS maps in the alpha and beta bands during passive and active movements with the affected hand and during bimanual passive and active movements (patient numbers 1, 2, and 3). Blue indicates maximal ERD. The *t*-test was applied individually for each patient in order to compare the ERD/ERS map of each patient to the mean ERD/ERS map of the controls (*p* < 0.1 (|*t*| > 1.895) indicated by (+)).

**Figure 2:**
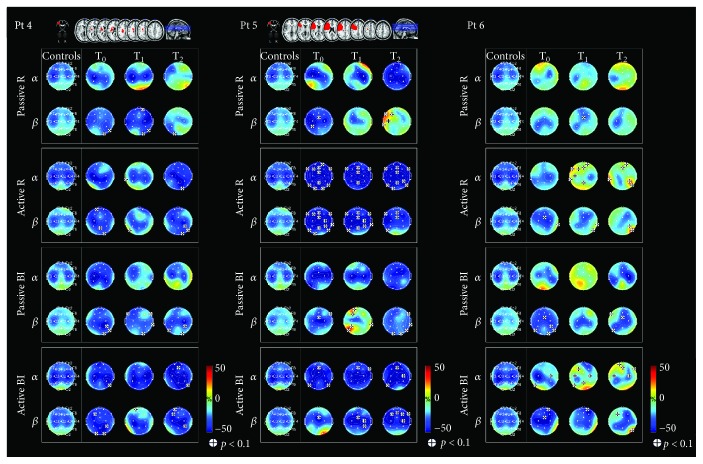
Lesions displayed on a magnetic resonance imaging brain template and topographic maps showing ERD/ERS values. ERD/ERS maps in the alpha and beta bands during passive and active movements with the affected hand and during bimanual passive and active movements (patient numbers 4, 5, and 6). Blue indicates maximal ERD. The *t*-test was applied individually for each patient in order to compare the ERD/ERS map of each patient to the mean ERD/ERS map of the controls (*p* < 0.1 (|*t*| > 1.895) indicated by (+)).

**Figure 3:**
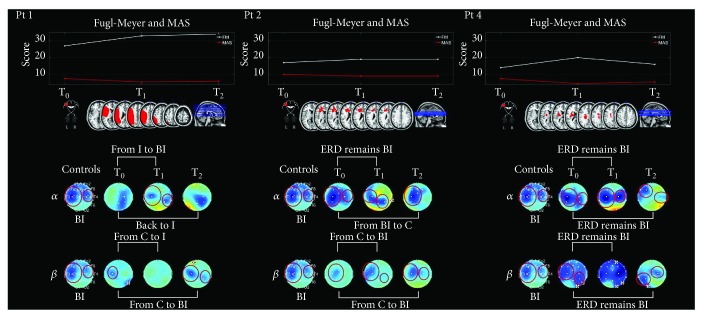
Fugl-Meyer and Modified Ashworth Scale scores, stroke lesions, and topographic maps showing ERD/ERS values during passive movement of the affected hand are reported for three representative patients (numbers 1, 2, and 4). Blue indicates maximal ERD. BI: bilateral ERD; C: ipsilesional ERD; I: contralesional ERD.

Five of the 7 patients were affected by moderate-to-severe upper limb paresis, and 2 by mild paresis (numbers 5 and 6). All 7 patients presented upper limb spasticity, 5 of which at the wrist (numbers 1, 2, 3, 4, and 7). All patients completed the R-BAT. No adverse events were reported. The regions of the brain that have sustained damage were summarized in Table 1. Stroke location was cortical-subcortical in all patients. However, brain lesion size and location varied among patients. The most damaged areas were the supplementary motor area, primary motor cortex, the primary somatosensory cortex, retrosubicular area, pars orbitalis, part of the inferior frontal gyrus, dorsolateral prefrontal cortex, inferior frontal gyrus, fusiform gyrus, the most rostral part of the superior and middle temporal gyrus, and the angular gyrus. White matter tracts included the anterior limb of the internal capsule, fasciculus fronto-occipitalis superior, the superior longitudinal fasciculus, external capsule, and superior corona radiate. ROIs were displayed in Figures 1, 2, and 3 (for details, see Tables 4, 5, 6, 7, and 8 in Supplementary Materials available here).

### 3.1. Primary Outcome

A significant improvement in FM scale was noted over time (*p* = 0.008) (for details, see Figure in Supplementary Materials. A greater than 10% change in FM scores at both T_0_-T_1_ and T_0_–T_2_ was observed in 4 patients (57%) (numbers 1, 2, 4, and 7) (Table 2).

Post hoc comparisons revealed significant effects between T_0_ and T_2_ by 2.85 (95% CI, 1.5 to 4.66) (*p* = 0.002). No statistically significant differences were noted between T_0_ and T_1_ (Table 3).

### 3.2. Secondary Outcomes

A significant improvement in MI (*p* = 0.009) and MAS (*p* = 0.002) scores over time was seen. Improvement on the MI was recorded for five patients (71%) (numbers 1, 4, 5, 6, and 7) at T_1_ and was maintained at T_2_. No changes in performance on the MI were recorded for two patients (28%) (numbers 2 and 3). Post hoc comparison revealed significant changes on MAS between T_0_-T_1_ (*p* = 0.017) and T_0_–T_2_ (*p* = 0.017). No significant effects were observed on the ARAT, BI, and MI.

### 3.3. Neurophysiological Evaluation

One patient (number 7) was excluded from the EEG analysis because of artifacts during recording. ANOVA of the alpha and the beta relative powers showed significant main effects for the factor “channel” in both the alpha [F(2.98,14.88) = 12.68, *p* < 0.001] and the beta [F(1.78,8.92) = 6.5, *p* < 0.05] band. No significant main effects for the factors “time” and “task” were observed. Significant interactions between “time” and “channel” were also observed in the alpha [F(36,180) = 1.47, *p* < 0.05] and the beta [F(36,180) = 1.596, *p* < 0.05] range. In particular, significant differences were observed in O1 (T_0_ versus T_1_, *p* < 0.001), O2 (T_0_ versus T_1_, *p* < 0.01), T6 (T_0_ versus T_1_, *p* < 0.05), and 28 (T_1_ versus T_2_, *p* < 0.05) in alpha band and in F7 (T_0_ versus T_1_, *p* < 0.05) in beta band.

No mirror movements occurred during unimanual movements, as confirmed by EMG.

### 3.4. Passive Movement with the Affected Hand

Individual EEG results are summarized in Figures 1 and 2, which display lesions on a magnetic resonance imaging brain template and topographic maps showing ERD/ERS values. The first column represents the maps from healthy subjects (control), the other three represent individual maps at specific time points. Alpha and beta rhythm (first and second row, resp.) are reported for each task.

Bilateral alpha desynchronization was observed at T_0_ in all patients except for patient number 1 who showed only contralesional ERD and for patient number 5 who showed ipsilesional ERD. At T_1_, four different patterns of alpha ERD were observed: bilateral (patient numbers 1, 2, and 4), contralesional (patient number 5), ipsilesional (patient number 6), and anterior (patient number 3). At T_2_, bilateral ERD was detected in 1 patient (number 5), contralesional ERD in 2 patients (numbers 1 and 3), and ipsilesional ERD in 3 patients (numbers 2, 4, and 6).

Bilateral beta desynchronization was observed at T_0_ in all patients, except for 2 (numbers 1 and 2) who showed ipsilesional ERD. As compared with the controls, ERD was significantly decreased over the right temporal and occipital electrodes in 1 patient (number 4) and over C3 and electrode 28 in 1 patient (number 5). At T_1_, the ERD pattern remained bilateral and different from the controls in the right temporal and occipital electrodes in 1 patient (number 4) and ipsilesional in 1 patient (number 2); anterior ERD was observed in 1 patient (number 1) and ipsilesional ERD in 3 patients (numbers 3, 5, and 6). As compared with the pattern observed in the controls, significant ERD over electrode 28 was noted in 1 patient (number 6). Three beta ERD patterns were observed at T_2_: (i) ipsilesional ERD in 3 patients (numbers 1, 2, and 6); (ii) bilateral ERD in 1 patient (number 4) with significant predominance over the ipsilesional side; and (iii) significant ipsilesional synchronization was noted in 1 patient (number 5) and significant ERD over anterior and central areas in 1 patient (number 3). Fugl-Meyer scores, stroke lesions, and topographic maps showing ERD/ERS values during passive movement of the affected hand are reported for three representative patients (numbers 1, 2, and 4) in Figure 3.

### 3.5. Active Movement with the Affected Hand

Three different patterns of alpha desynchronization were observed at T_0_: (i) bilateral ERD in 3 patients (numbers 2, 4, and 5); (ii) contralesional ERD in 2 patients (numbers 1 and 6); and (iii) ERD in central-posterior regions in 1 patient (number 3). These patterns remained unchanged at T_1_. The distribution was maintained at T_2_ in 4 patients (numbers 1, 2, 4, and 5), though widespread desynchronization was observed in 2 of them (numbers 4 and 5), with significant differences over the frontal regions as compared with the controls. Marked ERD on the contralesional side was observed in 1 patient (number 3). ERD became ipsilesional in 1 patient (number 6), with strong synchronization over the frontal electrodes and over T3, P4, and T6, as compared with the controls.

Three different patterns of beta ERD were observed at T_0_: (i) bilateral in 4 patients (numbers 2, 4, 5, and 6), with predominance over the ipsilesional side in 1 patient (number 2); (ii) there was a significant difference in central-posterior ERD in 1 patient (number 1) as compared with the controls, and (iii) in anterior-posterior ERD in 1 patient (number 3). These patterns remained unchanged at T_1_. Desynchronization became more ipsilesional to movement in 2 patients (numbers 2 and 5) at T_2_.

### 3.6. Bimanual Passive Movement

At T_0_, bilateral alpha ERD was found in 5 patients (numbers 1, 2, 4, 5, and 6), more localized over C4 in 1 patient (number 1). One patient (number 3) showed central-parietal ERD. At T_1_, bilateral desynchronization was maintained in 2 patients (numbers 2 and 5); ERD was localized at P4 in 2 patients (numbers 1 and 3), and over left central-anterior area in 1 patient (number 4), while ERS at P3 was observed in 1 patient (number 6). At T_2_, a bilateral pattern was observed in all patients. One patient (number 3) showed a more localized ERD over C4.

Two patterns of beta ERD were observed at T_0_: (i) bilateral in 4 patients (numbers 1, 4, 5, and 6), with significant predominance over C4 in 1 patient (number 4) and over the right posterior region in 1 patient (number 6), as compared with the controls and (ii) anterior-posterior ERD in 2 patients (numbers 2 and 3). A strong ERS was observed in 1 patient (number 3) over frontal areas and over right motor area. Four different patterns were found at T_1_: (i) bilateral ERD in 3 patients (numbers 1, 4, and 6); (ii) significant ERD over the right SM in 1 patient (number 3); (iii) ERD over C3 and significant ERS over P3 in 1 patient (number 5); and (iv) ERD over F3, 28, and C3 in 1 patient (number 2). Bilateral ERD was found in all patients at T_2_, with significant predominance over P4 in 3 patients (numbers 1, 3, and 4) and strong ERD localized over C3 in 1 patient (number 5).

### 3.7. Bimanual Active Movement

At T_0_, bilateral alpha ERD was found in 4 patients (numbers 1, 2, 4, and 5) and it was significantly localized over C3 in 1 patient (number 1); ERD over the left SM was observed in 2 patients (numbers 3 and 6). The alpha maps at T_1_ showed bilateral ERD in these 2 patients, with ERD over the right SM in patient number 3 and significant ERD over the left SM in patient number 6, as compared with the controls. Two patterns were observed at T_2_: (i) bilateral ERD in 4 patients (numbers 2, 4, 5, and 6), which was more localized over the right side in 2 patients (numbers 2 and 5) and (ii) ERD over the right SM in 2 patients (numbers 1 and 3) differently from the controls.

At T_0_, bilateral beta ERD was found in 3 patients (numbers 4, 5, and 6), more localized over the mesial region in 1 patient (number 5) and over F3, P4, O1, and O2 in 1 patient (number 4). ERD over C3 was observed in 3 patients (numbers 1, 2, and 3). At T_1_, bilateral ERD was detected in all patients, with predominance over C4 in 3 patients (numbers 2, 5, and 6). Three different patterns were observed at T_2_: (i) bilateral ERD in 3 patients (numbers 4, 5, and 6), with predominance over the left central-posterior electrodes in 1 patient (number 6); (ii) ERD over the left SM in 2 patients (numbers 1 and 2); and (iii) ERD over the right frontal-central electrodes in 1 patient (number 3).

### 3.8. Association between ERD/ERS Modulation and Clinical Scales

**Figure 4:**
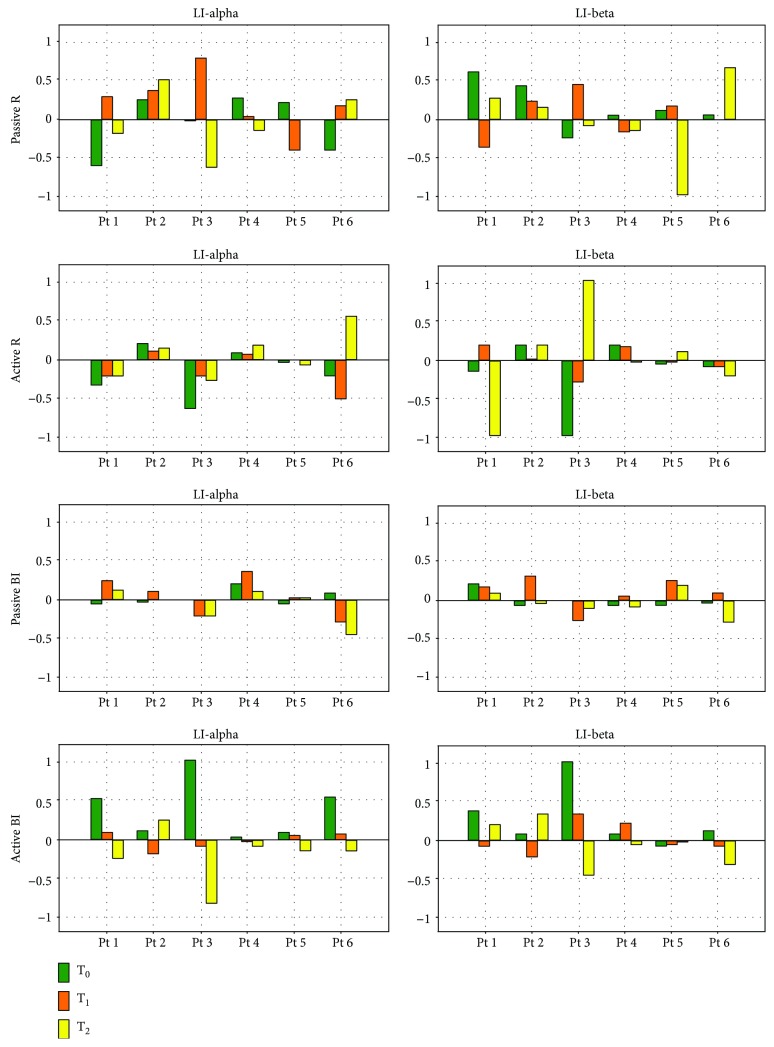
Laterality index (LI) in alpha and beta bands at each evaluation time point (T_0_, T_1_, and T_2_). LI was calculated considering contralateral (ipsilesional) ERD as the C3 ERD value over the left sensorimotor area and ipsilateral (contralesional) ERD as the C4 ERD over the right sensorimotor area. LI > 0 indicates contralateral ERD and LI < 0 indicates ipsilateral ERD.

No significant correlation was observed between LI and clinical scales (MAS and FM) at each evaluation time point. The only statistically significant correlation between ERD/ERS and clinical scales was for the passive bimanual motor task, where beta ERD over left sensorimotor area was positively correlated with MAS at T_2_ (*R* = 0.8459, *p* < 0.05). Nevertheless, EEG patterns and LI changes related to FM and MAS changes were observed over time. At T_0_, the majority of patients showed bilateral or contralesional activation of the primary sensorimotor cortex on alpha band during passive movement of the affected hand, as shown also by LI (Figure 4). Three patients (numbers 2, 4, and 5) showed ipsilesional activation. After training, desynchronization moved from contralesional to ipsilesional (patient numbers 1 and 6), from bilateral to ipsilesional (patient number 3), and from ipsilesional to bilateral (patient number 4) or to contralesional (patient number 5), as quantified by LI. The pattern remained ipsilesional in patient 2. These changes paralleled the improvements in the FM scores (Figure 3). No change in FM scores was noted in patient number 3, as confirmed by the anterior-posterior ERD. Regarding beta band, the 3 patients (numbers 4, 5, and 6) who showed bilateral activation before training moved to ipsilesional activation after training, as demonstrated also by the improvement in the MAS score at the wrist, in patient 3 desynchronization moved to contralesional to ipsilesional. At T_2_, patient number 1 had values close to the pretreatment ones as shown by neurophysiological evaluation in alpha band, while an improvement was noted in 2 patients (numbers 4 and 6) as shown by both clinical and neurophysiological evaluation.

During active movement of the affected hand, neurophysiological results in alpha band remained unchanged from T_0_ to T_2_ in 4 patients (bilateral ERD in numbers 2, 4, and 5 and contralesional in number 1), and a shift from contralesional to ipsilesional ERD was noted in 1 patient (number 6), as confirmed (except for patient number 1) by clinical evaluation, where FM score improved from T_0_ to T_2_, and by LI changes. Modification from T_0_ to T_2_ in beta band desynchronization was observed in 3 patients in which ERD shifted from bilateral to the ipsilesional side (numbers 2 and 5) and to contralesional to ipsilesional side (number 3).

## 4. Discussion

The main finding of our study was a reduction in upper limb spasticity after passive robot-assisted bilateral arm training. This effect was associated, albeit not significantly, with changes in cortical oscillatory activity, as demonstrated by the ERD/ERS maps and LI.

The most common deficit after stroke is hemiparesis of the contralesional UL [55]. However, the manifestation of UL motor impairment includes also muscle weakness, impaired motor control, deficits in somatic sensations, pain, and changes in muscle tone [55]. Poststroke spasticity is a disabling symptom affecting 17 to 43% of patients in the chronic stages of the illness [56]. It is now clear that spasticity encompasses a broad spectrum of clinical conditions, the so-called spastic movement disorder (SMD) [57]. Interdisciplinary complex rehabilitation interventions represent the mainstay of poststroke care [6]. Rehabilitation procedures, in particular, play a pivotal role in the management of SMD [58].

So far, limited knowledge is available to describe what best represents the optimum rehabilitative procedure. From a theoretical point of view, the UL rehabilitation program should include motor rehabilitation, multisensory interaction, hemispheric subspecialization in motor activity, and electrical brain stimulation [59]. Based on the current level of evidence, a decisional tree for UL rehabilitation has been proposed as a clinical tool for selecting a specific patient's intervention [55]. According to the stage of stroke, the presence of hand movements and the presence of spasticity-specific rehabilitation approaches and adjuvant techniques have been recommended [55]. Muscle strengthening exercises, treatment of spasticity, constraint-induced movement therapy, and mirror therapy have been recommended as a main rehabilitation intervention. However, most of these procedures cannot be performed in severely impaired patients. To this end, technology-supported training can overcome difficulties and deliver high-dosage and high-intensity training even in severely impaired patients [60]. Coupling the bilateral approach to robot-assisted therapy may be relevant in the rehabilitation of patient unable to perform active tasks.

Passive robot-assisted BAT has been shown to improve upper limb motor function in subacute and chronic stroke patients [11, 13, 19, 20, 61]. Moreover, two studies have shown positive training effects on spasticity [19, 20] though neither included neurophysiological measures. A full review of the pathophysiology of spasticity after stroke is beyond this perspective. Nevertheless, the overall evidence that the hyper excitable stretch reflexes may depend on imbalance of supraspinal inhibitory and excitatory inputs and on secondary soft tissue changes in the paretic limb has been established [62]. From a clinical point of view, spasticity in patients with upper motor neuron syndrome can be divided into two components. On the one hand, it can be mediated by the stretch reflex, which corresponds to spasticity itself. On the other hand, hypertonia can depend on soft tissue changes, which are referred as “nonreflex hypertonia.” Limb mobilization in patients with upper motor neuron syndrome is then essential to prevent and treat these two components [62].

Our findings are shared by a previous study by Hesse [20] and for the first time suggest that training effects could parallel specific changes in EEG power. Before training, passive movement of the affected hand resulted in bilateral or contralesional alpha and beta desynchronization of the primary sensorimotor cortex in the majority of patients, as quantified by LI (Figure 4).

After training, desynchronization shifted from bilateral to ipsilesional sensorimotor areas, and active movement of the affected hand shifted from contralesional to ipsilesional sensorimotor areas, as compared with pretreatment assessment. No significant correlation was observed between LI or ERD/ERS and clinical scales, but a marked trend was detected over time: changes in clinical scales, associated to a good recovery, paralleled with changes in LI values, which evidence modifications of ERD from contralesional to ipsilesional side.

Why R-BAT supports the observed findings remains a challenging question. The specific features of the training, in terms of type (passive, intensive, and repetitive) and duration (7 weeks), may have provided strong proprioceptive cueing by strengthening the neural pathways involved in upper limb function [63]. There is converging evidence that training protocols consisting of both active and passive movements (with or without visual feedback) tend to be more beneficial than passive movement alone in functional and neurophysiological outcomes [63, 64]. However, there is initial evidence suggesting that passive training may induce cortical reorganization, providing evidence for the notion that proprioceptive training could improve motor function [63]. Passive movements are a form of sensory stimuli (mainly proprioceptive input) that can activate the primary motor area (M1) and the primary somatosensory area (S1) through two mechanisms. The one is based on overlapping of the M1 and S1 maps, while the other is based on the fact that M1 receives somatosensory input directly from the thalamus [65]. This suggests that proprioceptive inputs are part of the motor control network during both the preparation and the execution of movement [63, 66, 67]. Robots might increase the sensorimotor experience by providing novel patient-environment interactions through passive and passive-mirrored repetitive training [68]. Note that training regimens lasting 6 weeks or longer tend to yield positive results [63].

The execution of bilateral pattern of movements might amplify these effects. In healthy controls, active-passive bilateral patterns of coordinated movement performed for 15 minutes disinhibit the M1 ipsilesional to the assisted upper limb and facilitate its excitability for at least 30 minutes [16]. In people with chronic stroke, daily application of active-passive R-BAT followed by motor practice was found to lead to a greater improvement in upper limb function as compared with motor practice alone [11]. These effects were associated with an increase in ipsilesional corticomotor excitability [11]. Passive R-BAT may facilitate cortical neural plasticity by two mechanisms. One consists of the simultaneous activation of both hemispheres, which is thought to facilitate activation of the damaged hemisphere by reducing transcallosal inhibition from the unaffected hemisphere. In this view, a rebalancing of interhemispheric inhibition would be enhanced [9, 11, 61, 69]. The other mechanism involves facilitation of the contralesional uncrossed corticospinal tract and spared indirect corticospinal pathways [22]. Finally, the training effects on muscle tone may be related to improvements in viscoelastic muscle properties [58].

To our knowledge, this is the first study that explores the effects of passive bilateral upper limb robot-aided rehabilitation by behavioral and EEG assessments in chronic stroke patients. So far, few studies have reported on the modulation of EEG cortical activity during robot-assisted tasks [17, 18, 29, 70, 71] and none have evaluated the effects of passive R-BAT on spasticity. The major advances in this study compared to our previous research is the investigation of neural correlates of behavioral changes after robot-assisted training in chronic stroke patients. Building on the results from our previous EEG studies on healthy subjects, few major novel aspects can be highlighted. Firstly, the feasibility of the EEG setup and motor paradigm was generated by movements in highly standardized robot-assisted paradigm to evaluate cortical activity in clinical setting. It allows for testing brain activity during specific rehabilitation tasks in different populations [72]. Second, the patients were in the chronic stage after stroke, which minimized the influence of confounding effects due to spontaneous recovery. In addition, a 2-week baseline assessment was performed to ensure motor function stability. Third, no combined treatments were associated with robotic training. In most previous studies, multiple modalities were combined in one training protocol, making it difficult to single out the effects of one specific modality [73] and to explore the neural correlates of specific robot-assisted training [74]. Finally, the training effects were evaluated at 1-month follow-up. Within this perspective, robotic devices can serve as highly standardized and reliable tools to inform the design of evaluation protocols and provide new insights into the dynamics of cortical reorganization promoted by rehabilitation.

The main limitation of the present study is the small sample size. Moreover, the lack of homogeneity between brain lesion size and location would have precluded statistically significant results. The ERD/ERS maps of the patients shown were different among themselves, and each map was also different with the control group in different ways. The current results varied across patients and precluded to conclude. According to these preliminary results, future studies would enroll larger sample size, and patients would be stratified according to lesion features. It would allow discussing EEG power changes after specific robot-assisted upper limb training (i.e., active, passive, unilateral, and bilateral). Other limitations are the lack of follow-up beyond 1 month and the lack of a control group receiving conventional therapy. Moreover, control group should be age-matched, in order to compare it to a group of subjects affected by stroke. However, since the peak frequency of the mu wave increases with age until maturation into adulthood, when it reaches its final and stable frequency of 8–13 Hz [75], the age of the subjects should not significantly affect the EEG desynchronization process during movement. These limitations notwithstanding, specific training effects on functional upper limb recovery and EEG power could be identified, as previously discussed.

## 5. Conclusion

The findings from the present pilot study may have implications for upper limb rehabilitation after stroke. Recovery may benefit from passive R-BAT program even years after the stroke event. Furthermore, bilateral repetitive robot-assisted training programs may sustain improvement in upper limb functioning in chronically impaired stroke patients and induce specific changes in the sensorimotor network. We speculate that the reduction in spasticity may have facilitated EEG changes over the ipsilesional sensorimotor network. The utility of a bilateral repetitive robot-assisted program as an adjuvant to physical therapy warrants further consideration.

## Figures and Tables

**Table 1:** Demographic and clinical data.

Patient	Age	Gender	Hand preference (°)	Poststroke (months)	Type of stroke	Side of stroke	Postrehabilitation (months)	Lesion mapping analysis (Brodmann areas/white matter tracts)		ESS (0–100^∗^)
1	61	M	+15	78	H	L	6	F, T, P	48, 40, 39, 6, 44, 45, 3, 7, 22, 41, 2, 42, 4, 19, 43, 37, 21, 47, 9, 46, 1, 18, 23, 10/41, 27, 29, 25, 33, 23, 17, 47, 21, 5	69
2	60	M	+23	61	I	L	6	F, T	48, 32, 46, 24, 11, 25, 45/23, 3, 4, 35, 17, 43, 25, 33	65
3	74	M	−18	80	I	L	12	F, T, P, O	44, 48, 46, 6, 45, 9, 4, 3, 32, 19/25, 41, 24	69
4	64	M	+24	20	I	L	6	T	48/25, 4, 19, 33, 43, 17, 27, 23, 41, 5	70
5	49	M	−16	21	I	L	6	F, T, P	48, 45, 44, 6, 46, 43, 4, 3, 47, 32, 9, 38, 22, 10/25, 23, 41, 4, 33, 43, 17, 3, 35	82
6	49	M	−15	123	H	L	18	F, P		79
7	56	M	+24	83	I	R	6	F, T, P, O	48, 37, 19, 20, 21, 18, 40, 6, 39, 22, 3, 38, 4, 2, 44, 47, 7, 41, 42, 43, 17, 34, 45, 1, 11, 36, 30, 28, 35, 27/42, 34, 26, 32, 30, 28, 40, 22, 46, 4, 24	64
Mean SD	59 8.79			66.57 36.61			8.57 4.72			71.14 6.82

M: male; °Briggs and Nebes' laterality inventory; I: ischemic; H: hemorrhagic; R: right; L: left; SD: standard deviation; ESS: European Stroke Scale; F: frontal lobe; T: temporal lobe; P: parietal lobe; O: occipital lobe; (): range; ^∗^best performance. Brodmann areas and white matter tracts are reported in decreasing order according to the number of involved voxels.

**Table 2:** Changes in primary and secondary outcome scores.

Patient	Fugl-Meyer (0–66)			Action Research Arm Test (0–57)			Motricity Index (0–100)			UL MAS (0–16)			Barthel Index (0–100)		
	T_0_	T_1_	T_2_	T_0_	T_1_	T_2_	T_0_	T_1_	T_2_	T_0_	T_1_	T_2_	T_0_	T_1_	T_2_
1	27	33^∗^	34^∗^	2	2	2	39	47	47	7.5	5.5	6	90	90	90
2	17	19^∗^	19^∗^	0	0	0	33	33	33	10	9	9	90	90	90
3	33	33	34	16	16	16	65	65	65	6	4.5	4.5	85	85	85
4	14	20^∗^	16^∗^	0	0	0	28	44	39	7.5	4.5	5.5	95	100	100
5	61	63	64	33	53	55	72	84	76	3	1	1	80	85	90
6	50	54	53	27	35	41	76	99	92	1	0	0	100	100	100
7	14	17^∗^	16^∗^	2	2	2	39	44	44	8.5	6.5	7	90	90	90
Mean/median	30.86	34.14	33.71	11.43	15.43	16.57	50.29	59.43	56.57	7.5	4.5	5.5	90.00	91.43	92.14
SD/Q1–Q3	18.51	18.04	18.86	13.95	22.01	22.54	20.00	24.25	21.67	3–8.5	1–6.5	5.5–7	6.45	6.27	5.67

T_0_: baseline assessment; T_1_: after training; T_2_: 1-month follow-up; UL: upper limb; MAS: Modified Ashworth Scale; ^∗^change in Fugl-Meyer score greater than 10%; (): range of score; SD: standard deviation; Q1–Q3: 1st quartile to 3rd quartile.

**Table 3:** Within-group training effects on clinical outcome measures.

	Friedman's two-way analysis of variance T_0_–T_2_	Post hoc comparisons T_0_-T_1_		Post hoc comparisons T_0_–T_2_	
	*p* value	*p* value	95% CI Mean (LB, UP)	*p* value	95% CI Mean (LB, UP)
*Primary outcome*					
Fugl-Meyer (0–66^∗^)	0.008^∗^	0.027	3.28 (1.23, 5.33)	0.017°	2.85 (1.05, 4.66)
*Secondary outcomes*					
Action Research Arm Test (0–57^∗^)	n.s.	n.s.	4.00 (−3.08, 11.08)	n.s.	1.14 (−0.95, 3.24)
Motricity Index upper limb (0–100^∗^)	0.009^∗^	0.04	9.14 (1.28, 16.99)	0.04	6.28 (0.87, 11.69)
Barthel Index (0–100^∗^)	n.s.	n.s.	1.42 (−0.82, 3.68)	n.s.	2.14 (−1.49, 5.78)
Modified Ashworth Scale (0–16^∗^)	0.002^∗^	0.017°	−1.78 (−2.43, −1.13)	0.02	−1.5 (−1.87, −1.12)
Stroke Impact Scale (0–800^∗^)	n.s.	n.s.	11.18 (−19.19, 41.56)	n.s.	7.11 (−18.71, 32.94)

T_0_: baseline assessment; T_1_: after training; T_2_: 1-month follow-up; CI: confidence interval; LB: lower bound; UP: upper bound; n.s.: not significant. ^∗^ *p* value significant at ≤0.05; °*p* value significant at ≤0.025.

## Supplementary Materials

Supplementary Materials Figure: upper limb behavioral scores. Table 4: brain lesion mapping results in patient number 1. Table 5: brain lesion mapping results in patient number 2. Table 6: brain lesion mapping results in patient number 3. Table 7: brain lesion mapping results in patient number 4. Table 8: brain lesion mapping results in patient number 5. Table 9: brain lesion mapping results in patient number 7.
