# Supplementary material for: Quantification of Upper Limb Motor Recovery and EEG Power Changes after Robot-Assisted Bilateral Arm Training in Chronic Stroke Patients: A Prospective Pilot Study
Source: Neural Plast. 2018 Mar 26;2018:8105480. doi: 10.1155/2018/8105480 (PMC5892248; doi:10.1155/2018/8105480)
Supplement: Supplementary Materials — Figure: upper limb behavioral scores. Table 4: brain lesion mapping results in patient number 1. Table 5: brain lesion mapping results in patient number 2. Table 6: brain lesion mapping results in patient number 3. Table 7: brain lesion mapping results in patient number 4. Table 8: brain lesion mapping results in patient number 5. Table 9: brain lesion mapping results in patient number 7. [file 8105480.f1.docx]

**Supplementary Material**

**Figure**

**
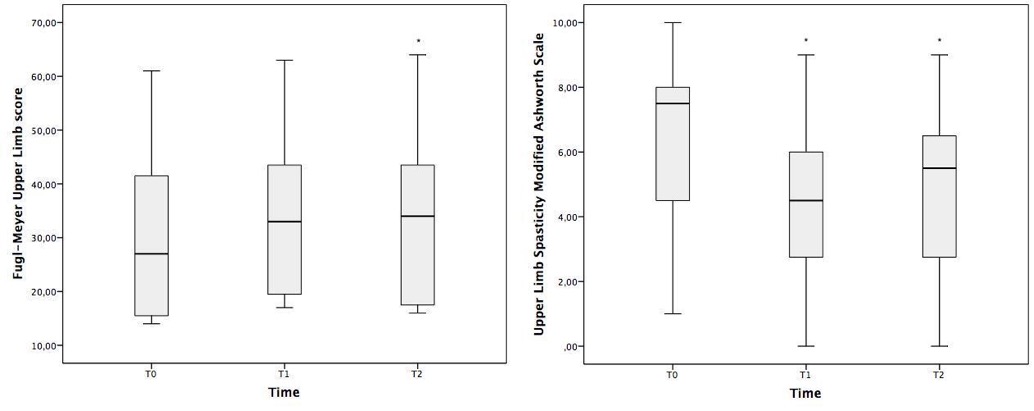
**

**Tables**

**Table 4.** Brain lesion mapping results in patient no. 1

| Brodmann areas | N. voxels | Anatomical  area | White tracts | N. voxels | Anatomical  area |
| --- | --- | --- | --- | --- | --- |
| 48 | 35163 | Retrosubicular area | 0 | 123022 | Unclassified |
| 0 | 13971 | White tracts | 41 | 5133 | Superior longitudinal fasciculus |
| 40 | 12491 | Supramarginal gyrus, | 27 | 1324 | Posterior corona radiate |
| 39 | 10113 | Angular gyrus | 29 | 1195 | Posterior thalamic radiations |
| 6 | 9614 | Supplementary Motor Area | 25 | 1049 | Superior corona radiate |
| 44 | 7375 | Pars opercularis, part of the inferior frontal gyrus and part of Broca's area | 33 | 685 | External capsule |
| 45 | 5508 | Pars triangularis, part of the inferior frontal gyrus and part of Broca's area | 23 | 469 | Anterior corona radiate |
| 3 | 5181 | Primary somatosensory cortex | 17 | 97 | Anterior limb of internal capsule |
| 7 | 4755 | Area parietalis superior | 47 | 96 | Corpus Callosum, Tapetum |
| 22 | 3527 | Superior temporal gyrus | 21 | 93 | Retrolenticular part of internal capsule |
| 41 | 3493 | Anterior transverse temporal gyrus | 5 | 49 | Corpus Callosum, Splenium |
| 2 | 3366 | Primary somatosensory cortex |  |  |  |
| 42 | 3282 | Posterior transverse temporal gyrus |  |  |  |
| 4 | 3260 | Primary Motor Cortex |  |  |  |
| 19 | 2855 | Peristriate Cortex |  |  |  |
| 43 | 2550 | Primary gustatory cortex |  |  |  |
| 37 | 2169 | Fusiform gyrus |  |  |  |
| 21 | 1992 | Middle temporal gyrus |  |  |  |
| 47 | 1460 | inferior frontal gyrus, pars orbitalis |  |  |  |
| 9 | 314 | Dorsolateral prefrontal cortex |  |  |  |
| 46 | 307 | Dorsolateral prefrontal cortex |  |  |  |
| 1 | 181 | Primary Somatosensory Cortex |  |  |  |
| 18 | 157 | Secondary visual cortex |  |  |  |
| 23 | 101 | posterior cingulate cortex |  |  |  |
| 10 | 27 | Anterior prefrontal cortex |  |  |  |

**Table 5.** Brain lesion mapping results in patient no. 2

| Brodmann areas | N. voxels | Anatomical  area | White tracts | N. voxels | Anatomical  area |
| --- | --- | --- | --- | --- | --- |
| 48 | 4579 | Retrosubicular area | 0 | 5620 | Unclassified |
| 0 | 3837 | White matter | 23 | 2809 | Anterior corona radiate |
| 32 | 908 | Dorsal anterior cingulate cortex | 3 | 724 | Corpus callosum |
| 46 | 253 | Dorsolateral prefrontal cortex | 4 | 319 | Corpus callosum |
| 24 | 104 | Ventral anterior cingulate cortex | 35 | 150 | Cingulate |
| 11 | 10 | Orbitofrontal area | 17 | 44 | Anterior limb of internal capsule |
| 25 | 5 | Subgenual area - Ventromedial prefrontal cortex | 43 | 26 | Fascicolus fronto-occipitalis superior |
| 45 | 5 | Pars triangularis, part of the inferior frontal gyrus and part of Broca's area | 25 | 6 | Superior corona radiate |
|  |  |  | 33 | 3 | External capsule |

**Table 6.** Brain lesion mapping results in patient no. 3

| Broadmann areas | N. voxels | Anatomical  area | White tracts | N. voxels | Anatomical  area |
| --- | --- | --- | --- | --- | --- |
| 44 | 10792 | Pars opercularis, part of the inferior frontal gyrus and part of Broca's area | 0 | 48200 | Unclassified |
| 48 | 8136 | Retrosubicular area | 25 | 246 | Superior corona radiate |
| 46 | 7942 | Dorsolateral prefrontal cortex | 41 | 121 | Superior longitudinal fasciculus |
| 6 | 7821 | Supplementary motor area | 24 | 12 | Anterior corona radiate |
| 45 | 5529 | Pars triangularis, part of the inferior frontal gyrus and part of Broca's area |  |  |  |
| 0 | 4559 | White matter |  |  |  |
| 9 | 1833 | Dorsolateral prefrontal cortex |  |  |  |
| 4 | 1244 | Primary Motor Cortex |  |  |  |
| 3 | 714 | Primary Somatosensory Cortex |  |  |  |
| 32 | 6 | Dorsal anterior cingulate cortex |  |  |  |
| 19 | 3 | Associative visual cortex |  |  |  |

**Table 7.** Brain lesion mapping results in patient no. 4

| Brodmann areas | N. voxels | Anatomical  area | White tracts | N. voxels | Anatomical  area |
| --- | --- | --- | --- | --- | --- |
| 0 | 6882 | White matter | 0 | 4005 | Unclassified |
| 48 | 4171 | Retrosubicular area | 25 | 3660 | Superior corona radiate |
|  |  |  | 4 | 1166 | Corpus callosum |
|  |  |  | 19 | 613 | Posterior limb of internal capsule |
|  |  |  | 33 | 553 | External capsule |
|  |  |  | 43 | 316 | Fascicolus fronto-occipitalis superior |
|  |  |  | 17 | 235 | Anterior limb of internal capsule |
|  |  |  | 27 | 168 | Posterior corona radiate |
|  |  |  | 23 | 161 | Anterior corona radiate |
|  |  |  | 41 | 156 | Superior longitudinal fasciculus |
|  |  |  | 5 | 20 | Corpus Callosum Splenium |

**Table 8.** Brain lesion mapping results in patient no. 5

| Brodmann areas | N. voxels | Anatomical  area | White tracts | N. voxels | Anatomical  area |
| --- | --- | --- | --- | --- | --- |
| 48 | 24093 | Retrosubicular area | 0 | 57323 | Unclassified |
| 0 | 11133 | White matter | 25 | 5016 | Superior corona radiate |
| 45 | 9567 | Pars triangularis, part of the inferior frontal gyrus and part of Broca's area | 23 | 1943 | Anterior corona radiate |
| 44 | 7933 | Pars opercularis, part of the inferior frontal gyrus and part of Broca's area | 41 | 1697 | Superior longitudinal fasciculus |
| 6 | 6668 | Supplementary motor area | 4 | 1022 | Corpus callosum |
| 46 | 2779 | Dorsolateral prefrontal cortex | 33 | 441 | External capsule |
| 43 | 2080 | Primary gustatory cortex | 43 | 210 | Fascicolus fronto-occipitalis superior |
| 4 | 1568 | Primary Motor Cortex | 17 | 89 | Anterior limb of internal capsule |
| 3 | 1210 | Primary Somatosensory Cortex | 3 | 84 | Corpus callosum |
| 47 | 390 | Pars orbitalis, part of the inferior frontal gyrus | 35 | 78 | Cingulate |
| 32 | 382 | Dorsal anterior cingulate cortex |  |  |  |
| 9 | 57 | Dorsolateral prefrontal cortex |  |  |  |
| 38 | 36 | Temporopolar area |  |  |  |
| 22 | 7 | Superior temporal gyrus |  |  |  |
| 10 | 1 | Anterior prefrontal cortex |  |  |  |

**Table 9.** Brain lesion mapping results in patient no. 7

| Brodmann areas | N. voxels | Anatomical  area | White tracts | N. voxels | Anatomical  area |
| --- | --- | --- | --- | --- | --- |
| 48 | 52200 | Retrosubicular area | 0 | 293412 | Unclassified |
| 37 | 27768 | Fusiform gyrus | 42 | 5743 | Superior longitudinal fasciculus |
| 0 | 27251 | White matter | 34 | 2691 | External capsule |
| 19 | 26957 | Associative visual cortex | 26 | 2576 | Superior corona radiate |
| 20 | 21783 | Inferior temporal gyrus | 30 | 1592 | Talamus posterior |
| 21 | 20281 | Middle temporal gyrus | 28 | 668 | Posterior corona radiate |
| 18 | 19419 | Secondary visual cortex | 40 | 665 | Fornix |
| 40 | 14783 | Supramarginal gyrus | 22 | 488 | Pars retrolenticularis of internal capsule |
| 6 | 14053 | Supplementary motor area | 46 | 376 | Fasciculus uncinatus |
| 39 | 13140 | Angular gyrus | 4 | 146 | Corpus callosum |
| 22 | 11756 | Superior temporal gyrus | 24 | 70 | Anterior corona radiate |
| 3 | 10419 | Primary Somatosensory Cortex |  |  |  |
| 38 | 7804 | Temporopolar area |  |  |  |
| 4 | 6325 | Primary Motor Cortex |  |  |  |
| 2 | 5792 | Primary Somatosensory Cortex |  |  |  |
| 44 | 5227 | Pars opercularis, part of the inferior frontal gyrus and part of Broca's area |  |  |  |
| 47 | 4481 | Pars orbitalis, part of the inferior frontal gyrus |  |  |  |
| 7 | 4179 | Visuo-Motor Coordination |  |  |  |
| 41 | 3425 | Auditory cortex |  |  |  |
| 42 | 3264 | Auditory cortex |  |  |  |
| 43 | 3225 | Primary gustatory cortex |  |  |  |
| 17 | 2888 | Primary visual cortex |  |  |  |
| 34 | 1772 | Dorsal entorhinal cortex |  |  |  |
| 45 | 699 | Pars triangularis, part of the inferior frontal gyrus and part of Broca's area |  |  |  |
| 1 | 654 | Primary Somatosensory Cortex |  |  |  |
| 11 | 449 | Orbitofrontal area |  |  |  |
